# Prediction of non-recovery from ventilator-demanding acute respiratory failure, ARDS and death using lung damage biomarkers: data from a 1200-patient critical care randomized trial

**DOI:** 10.1186/s13613-016-0212-y

**Published:** 2016-11-21

**Authors:** Jens-Ulrik S. Jensen, Theis S. Itenov, Katrin M. Thormar, Lars Hein, Thomas T. Mohr, Mads H. Andersen, Jesper Løken, Hamid Tousi, Bettina Lundgren, Hans Christian Boesen, Maria E. Johansen, Sisse R. Ostrowski, Pär I. Johansson, Jesper Grarup, Jørgen Vestbo, Jens D. Lundgren, For The Procalcitonin And Survival Study (PASS) Group, M. Steensen, K. Thornberg, M. Bestle, D. Strange, A. Ø. Lauritsen, P. Søe-Jensen, N. Reiter, N. E. Drenck, P. Fjeldborg, Z. Fox, J. Kjær, D. Kristensen, M. B. Rasmussen, C. S.v. Hallas, M. Zacho, C. Østergaard, P. L. Petersen, S. Hougaard, T. Mantoni, L. Nebrich, A. Bendtsen, L. H. Andersen, F. Bærentzen, Andreas Eversbusch, B. Bømler, R. Martusevicius, T. Nielsen, P. M. Bådstøløkken, U. Grevstad, P. Hallas, A. Lindhardt, T. Galle, K. Graeser, E. Hohwu-Christensen, P. Gregersen, L. M. Pedersen, I. Rye, J. Cordtz, K. R. Madsen, P. R. C. Kirkegaard, L. Findsen, L. H. Nielsen, D. H. Pedersen, J. H. Andersen, C. Albrechtsen, A. Jacobsen, T. Jansen, A. G. Jensen, H. H. Jørgensen, M. Vazin, L. Lipsius, M. Skielboe, B. Thage, C. Thoft, M. Uldbjerg, E. Anderlo, M. Engsig, F. Hani, R. B. Jacobsen, L. Mulla, U. Skram, T. Waldau, T. Faber, B. Andersen, I. Gillesberg, A. Christensen, C. Hartmann, R. Albret, D. S. Dinesen, K. Gani, M. Ibsen, J. A. Petersen, P. Carl, E. Gade, D. Solevad, C. Heiring, M. Jørgensen, K. Ekelund, A. Afshari, N. Hammer, M. Bitsch, J. S. Hansen, C. Wamberg, T. D. Clausen, R. Winkel, J. Huusom, D. L. Buck, U. Grevstad, K. Lenz, P. Mellado, H. Karacan, J. Hidestål, J. Høgagard, J. Højbjerg, J. Højlund, S. Hestad, M. Østergaard, N. Wesche, S. A. Nielsen, H. Christensen, H. Blom, C. H. Jensen, K. Nielsen, N. G. Holler, C. D. Rossau, M. Glæemose, M. B. Wranér, C. B. Thomsen, B. Rasmussen, C. Lund-Rasmussen, B. Bech, K. Bjerregaard, L. Spliid, L. L. W. Nielsen, K. M. Larsen, M. Goldinger, D. Illum, C. Jessen, A. Christiansen, A. Berg, T. Elkmann, J. A. K. Pedersen, M. Simonsen, H. Joensen†, H. Alstrøm, C. Svane, A. Engquist

**Affiliations:** 1 CHIP/Department of Infectious Diseases, Rigshospitalet, University of Copenhagen, Blegdamsvej 9, Copenhagen Ø, Denmark; 2 Department of Clinical Microbiology, Copenhagen University Hospital, Hvidovre, Denmark; 3 Department of Anesthesia and Intensive Care, Copenhagen University Hospital, Hillerød, Denmark; 4 Department of Anesthesia and Intensive Care, Bispebjerg Hospital, Copenhagen University Hospital, Copenhagen, Denmark; 5 Department of Anesthesia and Intensive Care, Copenhagen University Hospital, Gentofte, Denmark; 6 Department of Anesthesia and Intensive Care, Copenhagen University Hospital, Glostrup, Denmark; 7 Department of Anesthesia and Intensive Care, Aarhus University Hospital, Aarhus, Denmark; 8 Department of Anesthesia and Intensive Care, Copenhagen University Hospital, Hvidovre, Denmark; 9 Department of Anesthesia and Intensive Care, Copenhagen University Hospital, Herlev, Denmark; 10 Centre for Thrombosis and Hemostasis, Rigshospitalet, Copenhagen University Hospital, Copenhagen Ø, Denmark; 11 Centre for Respiratory Medicine and Allergy, University South Manchester Hospital NHS Foundation Trust and University of Manchester, Manchester, UK

**Keywords:** Lung damage, Mechanical ventilation, Biomarkers, Personalized early intervention

## Abstract

**Background:**

It is unclear whether biomarkers of alveolar damage (surfactant protein D, SPD) or conductive airway damage (club cell secretory protein 16, CC16) measured early after intensive care admittance are associated with one-month clinical respiratory prognosis. If patients who do not recover respiratory function within one month can be identified early, future experimental lung interventions can be aimed toward this high-risk group. We aimed to determine, in a heterogenous critically ill population, whether baseline profound alveolar damage or conductive airway damage has clinical respiratory impact one month after intensive care admittance.

**Methods:**

Biobank study of biomarkers of alveolar and conductive airway damage in intensive care patients was conducted. This was a sub-study of 758 intubated patients from a 1200-patient randomized trial. We split the cohort into a “learning cohort” and “validating cohort” based on geographical criteria: northern sites (learning) and southern sites (validating).

**Results:**

Baseline SPD above the 85th percentile in the “learning cohort” predicted low chance of successful weaning from ventilator within 28 days (adjusted hazard ratio 0.6 [95% CI 0.4–0.9], *p* = 0.005); this was confirmed in the validating cohort. CC16 did not predict the endpoint. The absolute risk of not being successfully weaned within the first month was 48/106 (45.3%) vs. 175/652 (26.8%), *p* < 0.0001 (high SPD vs. low SPD). The chance of being “alive and without ventilator ≥20 days within the first month” was lower among patients with high SPD (adjusted OR 0.2 [95% CI 0.2–0.4], *p* < 0.0001), confirmed in the validating cohort, and the risk of ARDS was higher among patients with high SPD (adjusted OR 3.4 [95% CI 1.0–11.4], *p* = 0.04)—also confirmed in the validating cohort.

**Conclusion:**

Early profound alveolar damage in intubated patients can be identified by SPD blood measurement at intensive care admission, and high SPD level is a strong independent predictor that the patient suffers from ARDS and will not recover independent respiratory function within one month. This knowledge can be used to improve diagnostic and prognostic models and to identify the patients who most likely will benefit from experimental interventions aiming to preserve alveolar tissue and therefore respiratory function.

*Trial registration* This is a sub-study to the Procalcitonin And Survival Study (PASS), Clinicaltrials.gov ID: NCT00271752, first registered January 1, 2006

**Electronic supplementary material:**

The online version of this article (doi:10.1186/s13613-016-0212-y) contains supplementary material, which is available to authorized users.

## Background

Persistent ventilator-dependent respiratory failure is a major cause of death and prolonged admission to the intensive care unit [1]. Some patients suffer damage to the respiratory system during the intensive care course and do not recover the ability to breathe independently. Among other biomarkers of lung damage (e.g., surfactant protein A [2], soluble receptor for advanced glycation end products (sRAGE) [3] and Kerbs von Lungen [3]), surfactant protein D (SPD), produced by alveolar type II cells, has consistently shown to predict ARDS [2–5] and club cell secretory protein 16 (CC16, formerly known as Clara cell secretory protein-16) produced in the conductive airways has been claimed to have this ability also [3].

It is not known whether these biomarkers of alveolar damage and conductive airway damage, respectively, can predict one-month clinical respiratory prognosis. Current measures of respiratory failure (e.g., PaO_2_/FiO_2_ ratio) do not seem to capture which intensive care patients are at risk of persistent ventilator-dependent respiratory failure for one month or more.

Berlin ARDS criteria are highly useful in many patients for guiding treatment [6]; however, many patients with severe acute respiratory failure get excluded from the ARDS diagnosis because of lack of relevant radiological findings fulfilling the criteria, and some patients without alarming baseline PaO_2_/FiO_2_ ratios may have a very poor respiratory prognosis. Clinical focus in intubated patients has been largely centered on acute syndromes within the first week after admission. However, less attention has been given to one-month respiratory prognosis, a clinically important endpoint.

SPD, a 43-kDa member of the collectin superfamily, is a part of lung surfactant and has been shown to have an important role in the innate immune system by working as a pattern recognition molecule [7]. Additionally, SPD promotes chemotaxis of antigen-presenting cells [8] and influences the function of lymphocytes and neutrophils [9, 10]. In a murine transgenic model, mice overexpressing SPD seemed to be protected against lung damage in oxidative stress [11]. Increases in vascular phase SPD have been associated with acute lung injury and mortality [2].

Club cells are situated primarily in the terminal bronchioles [12]. The dominant secreted substance from these cells is CC16, a 16-kDa polypeptide involved in immunoregulation [13, 14]. CC16 decreases in chronic lung damage, and among smokers, CC16 is reduced proportionally to the number of pack years [15]. CC16 increases in plasma when airways are stimulated with endotoxin [16]; however, this has been disputed [17]. These two biomarkers represent ways of identifying alveolar damage (SPD) and conductive airway damage (CC16).

The primary aim of this study was, among patients with acute ventilator-dependent respiratory failure and complete follow-up, to determine whether initial profound alveolar damage (high SPD) or damage to conductive airways (high CC16) is associated with ARDS and has a detectable clinical impact one month after intensive care admittance, i.e., failure to successfully wean the patient from mechanical ventilation.

## Methods

### Study population

This was a multicenter observational study based on the cohort of ICU patients recruited into the 1200-patient randomized controlled trial, the Procalcitonin And Survival Study (PASS) from 2006 to 2011 [18]. Data were collected according to the Good Clinical Practice guidelines [19], and follow-up was complete on the measures in the case report forms. Regarding use of mechanical ventilation, this was followed up for all patients, also when patients were discharged from the recruiting ICU and admitted to another ICU. All participants gave written informed consent, and ethical approval for the study was granted by the Regional Ethics Committee for Copenhagen and Frederiksberg Communes in Denmark (references: KF 01 272 753, KF 11 297 287).

To be eligible for the PASS trial, patients had to be at least 18 years of age, enrolled within 24 h of admission to the intensive care unit, and have an expected intensive care admission length of at least 24 h. Patients with known highly elevated bilirubin levels (≥40 mg/dL) or triglycerides (≥1000 mg/dL) were not eligible because of interference with procalcitonin measurements. Patients were recruited from nine ICUs across Denmark. In the PASS trial, patients were randomized to a proactive antibiotic strategy guided by biomarker levels (high-exposure group, *n* = 604) vs. a standard antibiotic strategy (standard exposure group, *n* = 596). Two recent publications from the study describe the details [20, 21]. Mortality was unchanged by the antibiotic intervention [18]. The population for the current study was all primarily intubated patients from the PASS trial with sufficient serum for the bio-analysis at baseline (*n* = 758). The population was split into two subpopulations according to the geographical position of the site where the patient was recruited: a northern “learning cohort” (*n* = 405) and a southern “validating cohort” (*n* = 353). The study material includes a biobank with serum from all patients from all days in the ICU (*n* = 9915 samples). For simplicity, one month was defined as 28 days.

### Biomarker measurement

Serum levels of SPD and CC16 were measured at baseline in uniplicate by commercially available ELISA assays (BioVendor Research Products ELISA kit, Brno, Czech Republic). The lower limits of detection were 0.01 ng/ml in the SPD kit and 0.046 ng/ml in the CC16 kit, respectively. No measurements were below the detection rate. Samples above the calibration interval were diluted.

### Outcome assessment

Several endpoints were assessed in the analyses to explore the hypothesis:

1. “Not successfully weaning from mechanical ventilation within 28 days”: A patient who was weaned from respirator within 28 days and not re-intubated within the same period was considered to have been successfully weaned. Death within 28 days was considered a competing in these analyses.
2. “Alive and without ventilator ≥20 days within the first month”: Since death was frequent in this cohort (as in other ICU cohorts), we explored a combined “favorable” outcome of surviving and being independent of mechanical ventilation for ≥20 days.
3. ARDS according to the Berlin Criteria [6]: The endpoints were assessed using the case report form while in the initial ICU and post-ICU by finding all admissions to departments in Denmark; if the department was not able to administer mechanical ventilation, it was assumed that the patient was not using mechanical ventilation. If the department could administer mechanical ventilation, the patient chart was located and read to ascertain whether the patient received mechanical ventilation. Radiology was registered in the case report form. Bilateral opaque infiltrates not fully explained by other causes were considered suspicious for ARDS and were combined with the PaO_2_/FiO_2_ ratio in the definition. An ICU specialist judged whether the patient had ARDS (acute diagnosis in the case report form).

### Statistical analyses

Comparisons of continuous data were made using Student’s t tests and Mann–Whitney *U* tests where appropriate. Chi-square tests for equal proportions were used to test categorical variables; at small numbers, Fisher’s exact test was used. Time-to-event analyses were performed using Cox proportional hazards models and Kaplan–Meier plots with corresponding log-rank tests. For analyses of persistent respiratory failure, a competing risk model was applied to account for the effect of deaths during follow-up. Multivariable analysis of the primary endpoint was performed adjusting for known and suspected predictors of persistent respiratory failure: surfactant protein D ≥85th percentile (≥525.6 ng/mL), club cell secretory protein 16 ≥ 85th percentile (≥42.75 ng/mL), lowest quartile PaO_2_/FiO_2_ ratio (vs. quartile 2-4), APACHE II score (continuous), age (continuous), severe sepsis/septic shock (present vs. not), chronic obstructive lung disease (COPD, yes vs. no), Charlson’s score ≥2 (vs. <2), gender (male vs. female) and estimated glomerular filtration rate (continuous). “Alive and without ventilator ≥20 days within the first month” and “ARDS” were analyzed using multivariable logistic regression analysis.

A power calculation was done for the smallest cohort. Conditions for the calculation were as follows: sample size 353, conventional border for type I error (0.05), exposure variable present in 15% (biomarker level above 85% percentile), endpoint (successful weaning within 28 days) present in 55% patients in the high-level biomarker group, variance inflation factor of 0.2 and detection limit of hazard ratio in a Cox proportional hazards model of 0.60. This resulted in a power of 0.80. Power calculation was done using the Study Size 3.0 package, Creostat, Frölunda, Sweden. Statistical analyses were performed using SAS version 9.3 (SAS Institute Inc, Cary, NC) and “R” version 3.0.2 (The R-project, http://www.r-project.org/).

The study was initiated and run by doctors at our research department (CHIP, Rigshospitalet) and the participating intensive care units (ICU). All data belong to the PASS study group.

## Results

**Fig. 1:**
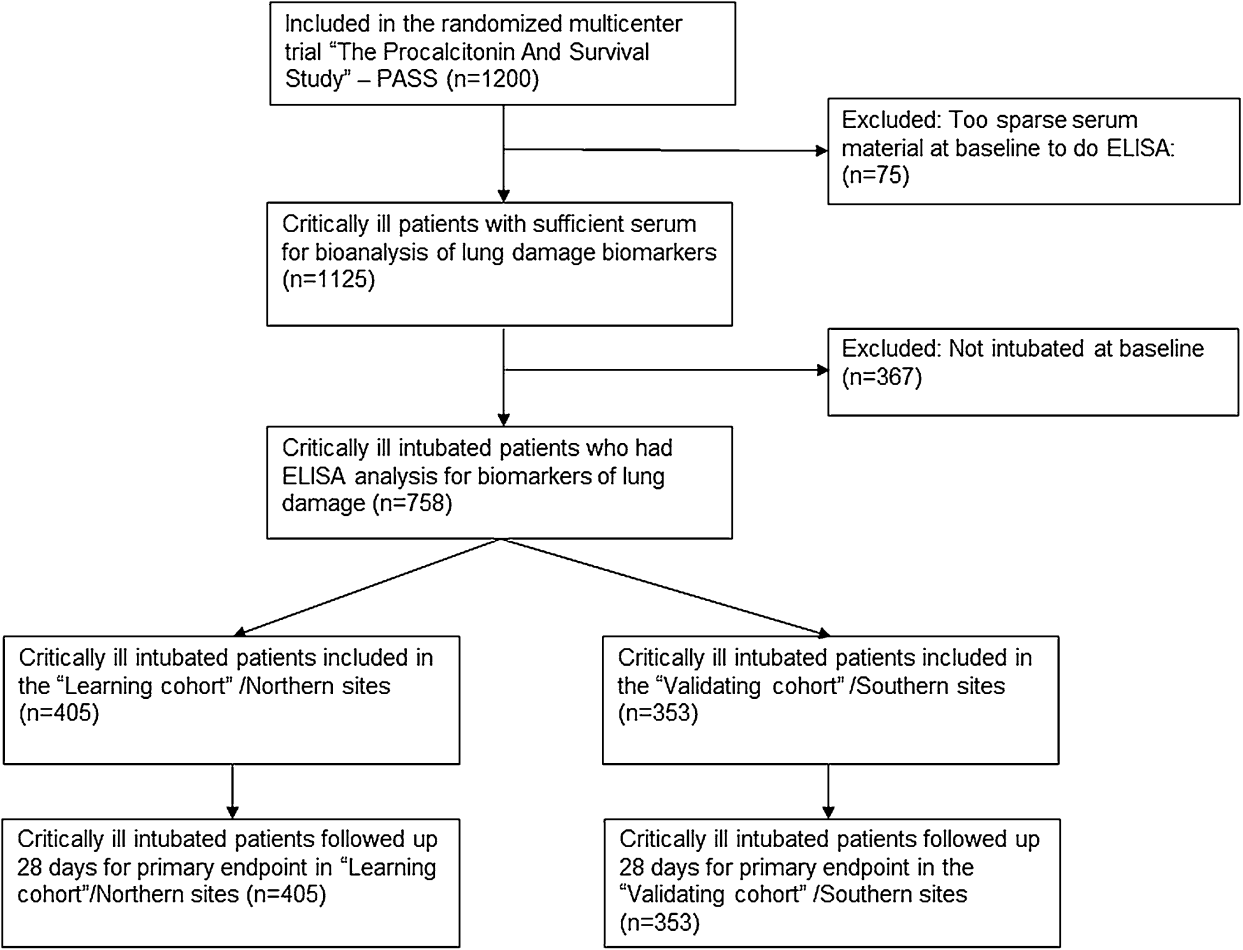
Flowchart of patients in the study. PASS: the Procalcitonin And Survival Study, a 1200-patient intensive care randomized trial [21]

Between January 2006 and June 2011, nine intensive care units in Denmark recruited and followed 1200 critically ill patients. Serum was collected for all patients on all ICU days; however, in 75 patients, not enough serum was available to perform ELISA analysis. Additionally 367 patients were not intubated at baseline; 758 patients were included in the current study (Fig. 1), 405 in the “learning cohort” and 353 in the “validating cohort.” The baseline characteristics in the two cohorts are given in Table 1; the most common primary reason for admission to the ICU was infection of different types, as given in Table 1.

**Table 1:** Baseline characteristics of the patients in the two cohorts

	Learning cohort (northern) *N* = 405	Validating cohort (southern) *N* = 353
Age (year) (median, IQR)	68 (59–77)	67 (57–75)
Apache II score (median, IQR)	21 (15–28)	19 (14–25)
Body mass index (kg/m^2^, median, IQR)	24.7 (22.8–27.7)	24.7 (22.2–27.7)
eGFR (ml/min/1.73 m^2^)	48.5 (26.4–78.9)	58.8 (33.5–93.5)
PaO2/FiO2 (Kpa, median, IQR)	19.4 (11.6–29.2)	21 (13.6–28.9)
Surfactant protein D (ng/mL, median, IQR)	133.5 (77.3–308.0)	112 (63.5–240.7)
Club cell secretory protein 16 (ng/mL, median, IQR)	25.4 (10.6–37.4)	20.2 (7.7–37.9)
Severe sepsis/septic shock, *n* (%)	158 (39.0)	149 (42.2)
Charlson’s comorbidity index, *n* (%)		
0	130 (32.1)	136 (38.5)
1	139 (34.3)	108 (30.6)
2	79 (19.5)	65 (18.4)
3	34 (8.4)	30 (8.5)
4	13 (3.2)	8 (2.3)
5	6 (1.5)	2 (0.6)
6	4 (1.0)	3 (0.9)
7	0 (0.0)	1 (0.3)
COPD, *n* (%)	81 (20.0)	75 (21.3)
Primary admittance cause/acute diagnosis category (%)		
Pneumonia	137 (33.8)	117 (33.1)
Abdominal sepsis	65 (16.1)	76 (21.5)
Sepsis of other cause	48 (11.9)	42 (11.9)
Heart failure	47 (11.6)	34 (9.6)
Thrombosis or bleeding	52 (12.8)	20 (5.7)
Other	56 (13.8)	64 (18.1)
Gender, *n* (%)		
Female	184 (45.4)	163 (46.2)
Male	221 (54.6)	190 (53.8)

y, years; IQR, interquartile range; COPD, chronic obstructive pulmonary disease; eGFR, estimated glomerular filtration rate—calculated by the Cockroft–Gault formula

Follow-up within 28 days after inclusion was complete both for the primary endpoint (weaning from ventilator-demanding respiratory) and for mortality in both cohorts.

**Fig. 2:**
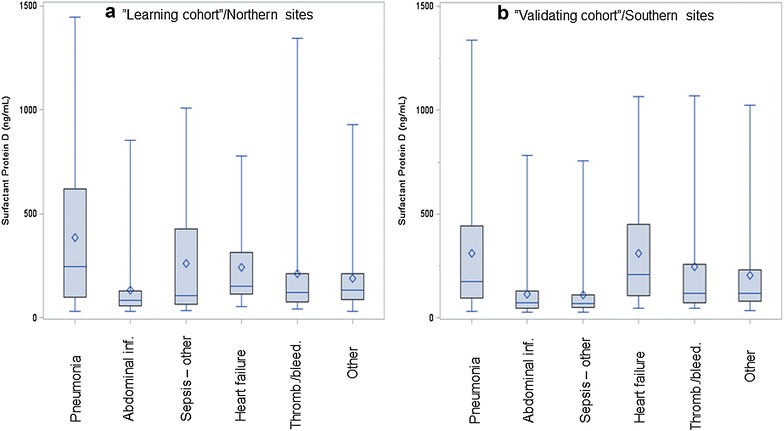
Surfactant protein D serum levels according to primary admission reason. **a** “Learning cohort”/northern sites. **b** “Validating cohort.” *Boxes* are medians and interquartile ranges. *Whiskers* are total range. *Rhombuses* are means

The highest levels of SPD were found in patients with pneumonia and heart failure as primary cause of ICU admission (Fig. 2); no differences were noted for CC16 between any diagnostic categories (not shown).

### Successful weaning from mechanical ventilation within 28 days

Failure to be successfully weaned from mechanical ventilation within 28 days was observed 105/405 (25.9%) in the “learning cohort” and 118/353 (33.4%) in the “validating cohort.” Death from all causes was considered a competing risk in all Cox regression analyses.

Patients with SPD above the 85th percentile (≥525.6 ng/mL) in the “learning cohort” were less likely to be successfully weaned from ventilator (adjusted HR 0.6 [95% CI 0.4–0.9], *p* = 0.0053); no association was found for CC16, and apart from SPD, only APACHE II score and severe sepsis/septic shock independently predicted reduced probability of successful weaning from mechanical ventilation within 28 days, Cox regression, as given in Table 2. After proposing and analyzing the 85th percentile cutoff in the “learning cohort”/northern cohort at 525.6 ng/mL SPD, all the above analyses were repeated in the “validating cohort”/southern cohort, using the cutoff established in the “learning cohort” (525.6 ng/mL). The signal was unchanged; high SPD independently predicted a reduced chance for the patient successfully to be weaned from mechanical ventilation within 28 days (adjusted HR 0.6 [95% CI 0.4–1.0], *p* = 0.046, Table 2).

**Table 2:** Predictors of successful weaning from mechanical ventilation within 28 days—multivariable competing risk Cox regression

	Learning cohort (northern) *N* = 405				Validating cohort (southern) *N* = 353			
	*P* value	Hazard ratio	95% CI for HR		*P* value	Hazard ratio	95% CI for HR	
			Lower	Upper			Lower	Upper
Surfactant protein D (≥85th percentile in “learning cohort,” ≥525.6 ng/mL vs. <525.6 ng/mL)	0.0053	0.60	0.42	0.86	0.046	0.64	0.42	0.99
Club cell secretory protein 16 (≥85th percentile in “learning cohort,” ≥42.8 ng/mL vs. <42.8 ng/mL)	0.50	0.89	0.66	1.22	0.81	0.96	0.67	1.37
PaO_2_/FiO_2_ (Q1 vs. Q2–Q4)	0.017	0.73	0.57	0.95	0.89	0.98	0.73	1.32
Apache II score (per score unit increase)	0.031	0.986	0.974	0.999	0.041	0.981	0.964	0.999
Age (per year increase)	0.84	1.00	0.992	1.010	0.17	0.993	0.984	1.003
Severe sepsis/Septic shock (vs. milder or no infection)	0.0014	0.66	0.51	0.85	0.0019	0.63	0.48	0.85
Charlson’s comorbidity index ≥2 vs. <2	0.046	1.29	1.00	1.67	0.58	1.08	0.81	1.46
Chronic obstructive pulmonary disease (yes vs. no)	0.36	0.87	0.64	1.18	0.71	0.94	0.67	1.31
Gender (male vs. female)	0.82	0.97	0.77	1.23	0.97	1.01	0.77	1.32
Estimated glomerular filtration rate (per ml)	0.61	1.00	0.999	1.002	0.048	1.001	0.998	1.004

Adjusted Cox regression risk estimates for known, suspected and explored predictors of successful weaning from ventilator within 28 days. Death from all causes was entered in the model as a competing risk

Q1, quartile 1; eGFR, ml/min/1.73 m^2^

Since the population was heterogenous, the above analyses were repeated in both cohorts after excluding patients considered to have a some degree of non-reversible cause of persistent respiratory failure (heart failure, severe neurological disease and chronic interstitial lung disease); the signal was unchanged: northern (learning) cohort (*n* = 346): SPD ≥ 525.6 ng/mL: adjusted HR 0.6 [95% CI 0.4–1.0, *p* = 0.028] and southern (validating) cohort (*n* = 300): SPD ≥ 525.6 ng/mL: adjusted HR 0.6 [95% CI 0.4–1.0, *p* = 0.050].

**Fig. 3:**
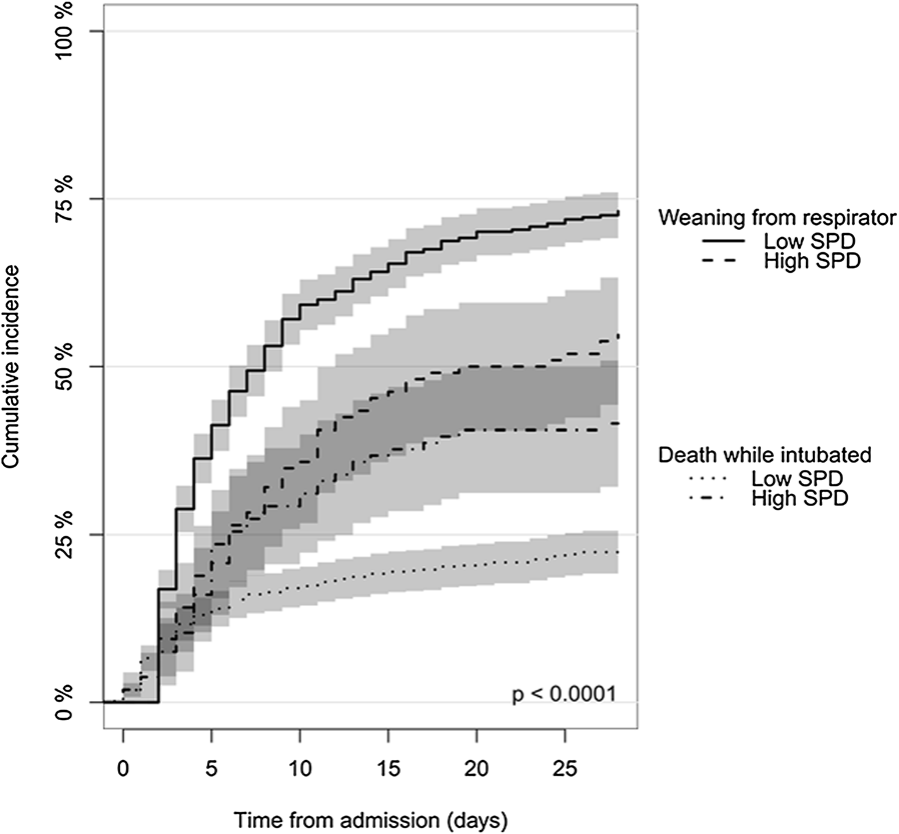
Cumulative incidence of successful weaning from respirator within 28 days after intensive care admission and death while intubated—total cohort (“learning”/northern cohort + “validating”/southern cohort). The two *upper curves* are regarding “successful weaning from respirator” (vs. still intubated at day 28); the two *lower curves* are regarding “dead while intubated” (vs. alive at day 28). Patients extubated <48 h at death were counted as “dead while intubated.” Patients extubated and alive at day 28 and those extubated ≥48 h at death were counted as successfully weaned from ventilator. *N* = 758. *Gray scales* are 95% CI. *SPD* surfactant protein D. “High SPD is >525.6 ng/mL

The hazard function for successful weaning from mechanical ventilation according to SPD serum level (the 15% patients with highest SPD, ≥525.6 ng/mL) for the entire cohort (*n* = 758) seemed not only to be different in the early course of the ICU admission; curves separate increasingly throughout the 28-day observation period, and patients with high SPD also had a substantially increased incidence of death while intubated, as shown in Fig. 3.

### Alive and without ventilator ≥20 days within the first month

**Fig. 4:**
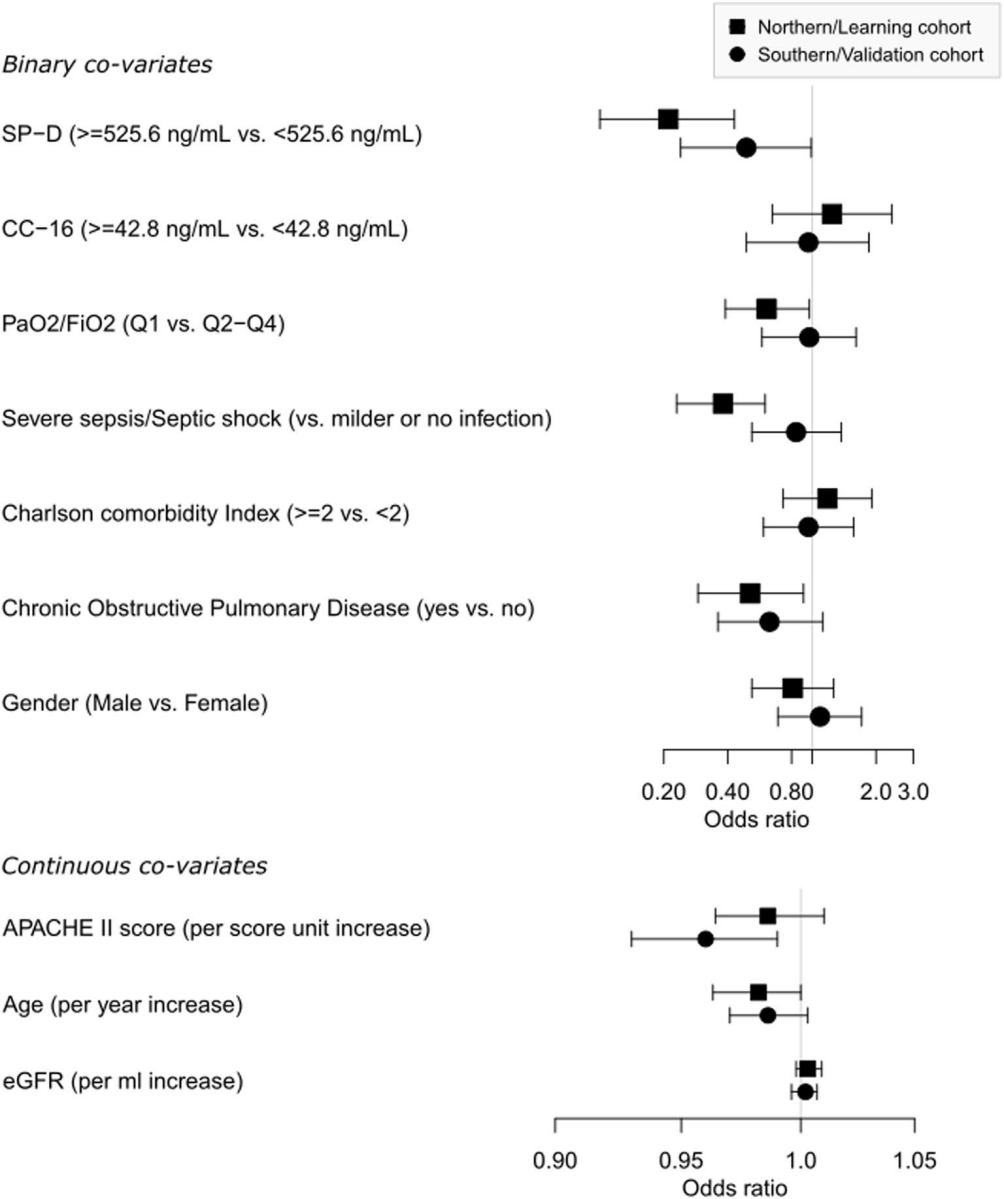
Adjusted odds ratios for the patient being “alive and without mechanical ventilation for ≥20 days within first 28 days after ICU admission.” All variables were entered in the same logistic regression model. The *graph* is separated to display odds ratios for both binary and continuous covariates. Cut points for SP-D and CC16 are equal to upper 15 percentile in the northern/learning cohort. *Boxes* and *whiskers* are odds ratio and 95% CIs, respectively

In the learning cohort, 211 of 405 patients were “alive and without ventilator ≥20 days within the first month.” In the validating cohort, this number was 154/353. In both cohorts, SPD ≥85th percentile (≥525.6 ng/mL) was an independent predictor of the patient being “alive and without ventilator ≥20 days within the first month” (Fig. 4).

### “Acute respiratory distress syndrome” according to Berlin criteria

In total, 31 (4.1%) patients fulfilled the ARDS Berlin definition at admission to the ICU. All patients were assessed for ARDS at baseline, but were not followed for this after discharge from the ICU. High SPD also independently predicted ARDS at baseline; however, CC16 did not predict these endpoints (Table 3).

**Table 3:** Acute respiratory distress syndrome^a^ according to Berlin criteria—multivariable logistic regression

	Learning cohort (northern) *N* = 405				Validating cohort (southern) *N* = 353			
	*P* value	Odds ratio	95% CI for OR		*P* value	Odds ratio	95% CI for OR	
			Lower	Upper			Lower	Upper
Surfactant protein D (≥85th percentile in “learning cohort,” ≥525.6 ng/mL vs. <525.6 ng/mL)	0.042^a^	3.4	1.0	11.4	0.003^a^	8.4	2.0	35.4
Club cell secretory protein 16 (≥85th percentile in “learning cohort,” ≥42.8 ng/mL vs. <42.8 ng/mL)	0.14	2.6	0.7	9.7	1.0	0.96	0.20	4.5
PaO_2_/FiO_2_ (Q1 vs. Q2-Q4)	0.076	2.9	0.9	9.1	0.001^a^	9.3	2.6	33.7
Apache II score (per score unit increase)	0.23	1.025	0.984	1.067	0.57	1.023	0.946	1.106
Age (per year increase)	0.56	1.015	0.965	1.067	0.26	0.974	0.93	1.02
Severe sepsis/septic shock (vs. milder or no infection)	0.19	2.1	0.68	6.8	0.06	3.6	0.96	13.6
Charlson’s comorbidity index ≥2 vs. <2	0.23	0.41	0.10	1.7	0.12	0.28	0.053	1.42
Chronic obstructive pulmonary disease (yes vs. no)	0.74	0.76	0.15	3.8	0.65	1.43	0.30	6.7
Gender (male vs. female)	0.32	0.56	0.18	1.74	0.67	1.30	0.40	4.2
Estimated glomerular filtration rate (per ml increase)	0.19	1.005	0.998	1.011	0.61	1.003	0.992	1.01

Adjusted logistic regression risk estimates for known, suspected and explored predictors of ARDS. Abbreviations: Q1: quartile 1

Biomarker levels were not known for radiologists and ICU physicians who diagnosed ARDS

OR, odds ratio

^a^All severities (mild, moderate, severe) of ARDS were counted

Patients with SPD ≥85th percentile (≥525.6 ng/mL) also had a high incidence of death from all causes as compared with patients with an SPD <85th percentile, as shown in Additional file 1: eFig 1.

## Discussion

The current study shows, in a well-defined cohort of 758 intubated critically ill patients, that profound alveolar damage at ICU admission, measured using SPD, increases the risk that the patient has ARDS, and substantially reduces the chance that patients can breathe on own conditions, without mechanical ventilation, one month after admission to the intensive care unit. Patients with high SPD also had a high risk of 28-day all-cause mortality. To acknowledge the competing risk of death, the main analysis was performed as a competing risk model. Additionally, an analysis of the endpoint “alive and without ventilator ≥20 days within the first month” was performed. The signal was unchanged; a high SPD was an independent predictor of this endpoint also. The effect was detected in a “learning cohort” and validated in a “validating cohort,” geographically separated. Results in both cohorts were robust to adjustment for nine other known or suspected predictors of respiratory failure [22, 23]. Additionally, the association is biologically plausible, since SPD is a documented part of the alveolar surfactant with immunological properties as a pattern recognition molecule, and serum SPD increases when alveolar damage emerges so a leakage to the blood can occur [7, 24, 25]. Almost half of the patients with high SPD were not capable of breathing on own conditions after one month was reached, compared to approximately one-fourth of those with SPD below the cutoff.

Opposite, CC16, a marker primarily produced in the conductive airways, did not predict the endpoint in any analysis.

The current results should be interpreted in context to the not easily recognized pathophysiological changes taking place in acute ventilator-dependent respiratory failure, sometimes leading to the clinical picture of acute respiratory distress syndrome (ARDS): diffuse damage to alveolar epithelial cells, including alveolar type II cells and vascular endothelium, breakdown of the basal membrane in the alveoli and consequent leakage of surfactant components to the blood. This is accompanied by hemorrhagic intra-alveolar deposition of platelets, protein and fibrin components eventually forming hyaline membranes [26].

Thus, our results indicate that patients who have an early increase in SPD more often progress into pathophysiological changes that are not easily reversible and changes that cause the phenotype of persistent ventilator-dependent respiratory failure. Our findings regarding PaO2/FiO2 ratio, an important acute parameter for the ICU physician, underline the need for pathophysiological markers like SPD to identify patients at early risk of persistent respiratory failure, since an unfavorable PaO_2_/FiO_2_ ratio in the lowest quartile was not a consistent predictor of poor respiratory prognosis after one month.

The knowledge provided by measuring SPD early does have important implications for predicting outcome, and it does increase the understanding of how and when the decisive pathophysiological steps leading to this feared clinical syndrome occur, but even more importantly, this knowledge could help at admission, identifying the most suitable candidates for trials applying experimental lung interventions in patients at high risk of developing persistent respiratory failure. Prone positioning has been demonstrated to be effective in patients with ARDS [27]; however, our results suggest that patients with a predicted high risk of persistent respiratory failure (i.e., highest SPD) should be enrolled in trials testing experimental lung interventions even before ARDS develops, in order to improve the prognosis [28]. ARDS awareness is of key importance in these vulnerable patients, so timely and effective interventions can be initiated. However, current reports show that ARDS is often not recognized, even when present [22]. Additionally, many patients who end up with persistent respiratory failure after one month may not have fulfilled ARDS criteria previous to this, and in some patients, an early warning by a biomarker, before ARDS develops, may provide a possibility for early intervention, even before clinical signs of poor prognosis can be realized. Thus, it seems reasonable to supplement increasing ARDS awareness with biomarkers of acute lung damage like SPD and probably others. In a rat model of ARDS, soluble receptor for advanced glycation end products (sRAGE) seemed to reflect alveolar type I cell injury, and this was also observed in humans [29, 30], and recently, it has been demonstrated that a strong negative correlation exists between alveolar fluid clearance rate and plasma sRAGE in a murine model as well as in humans [31]. Thus, sRAGE and SPD may provide complementary information on the pathophysiological changes taking place in the alveolar epithelium.

This knowledge does draw the attention to two issues of pivotal importance: i) that novel and experimental alveoli-protecting interventions should be instituted in a personalized manner—what works for one critically ill patient (with altered SPD, sRAGE and possibly other signals of profound lung damage) may not work for another patient with low SPD and no other significant signs of profound lung damage, and ii) that in patients with early signs of profound lung damage, experimental alveoli-protecting interventions should probably be tested in trials to reduce development of long-term respiratory failure.

### Strengths and limitations

The current study is an observational study with the built-in limitations this implies. Not all patients in the cohort had lung biomarker measurements (75 patients in the cohort had insufficient volume of serum, and we cannot expand our conclusions to the patients where biomarker measurements were not taken). We cannot document that awareness of ARDS was sufficient; however, this is not unique to our cohort [22]. Many factors that occur after the initial event that led to ICU admission may have influenced the development of persistent respiratory failure, which are tidal volumes, PEEP adjustments and the timing of these, transfusions, critical illness neuropathy and others. We could not control for all these factors, and especially timing of PEEP adjustments are, to some extent, still disputed. However, factors not present at baseline and therefore not possibly captured by the biomarkers at baseline will tend to underestimate the predictive power of the biomarkers. Optimally, biomarkers of lung damage should have been measured daily. The strengths of the study are (1) the relatively high sample size, (2) the complete follow-up for the endpoints due to good clinical practice-based clinical monitoring and follow-up based on the Danish hospital registers and (3) the completeness of data on a wide variety of clinical, biochemical, radiological and microbiological data in these patients.

Optimally, the results should be validated in another cohort of intubated ICU patients with complete follow-up on mechanical ventilation and mortality for 28 days, and where these two biomarkers were analyzed. Since we could not identify such a population by searching PubMed and EMBASE, we decided to split the cohort up according to geographical criteria to mimic that the validating cohort was another cohort. This strategy can be criticized, and our results should be further validated.

In summary, a high SPD level at baseline is associated with a very high absolute risk of the patient not being successfully weaned from mechanical ventilation within a month, and a high risk of dying while intubated. When adjusted for other predictors of respiratory failure, high SPD level at baseline independently predicts persistent respiratory failure during mechanical ventilation in critically ill patients. Acknowledging that ICU populations are often heterogenous and that other reasons may have accounted for some of the cases of persistent respiratory failure, we repeated all the main analyses while excluding patients with heart failure, chronic interstitial lung disease and severe neurological diseases; all these analyses confirmed the findings regarding the lung damage biomarkers.

The results from the current study indicate that high impact insults on the lungs, early in the ICU course, contribute substantially to the development of later persistent respiratory. Importantly, early SPD blood measurements can reveal evidence of such profound lung damage. This knowledge could be used in updated diagnostic and prognostic models regarding respiratory failure and ARDS in critically ill patients. If our results regarding SPD and prognosis can be verified in other cohorts, SPD measurements could be added to the ARDS criteria, since SPD seems to predict both respiratory prognosis and overall prognosis and, importantly, is linked to pathophysiology taking place in the alveoli. Additionally, SPD measurements could be used to select patients for trials on novel experimental lung interventions initiated in high-risk patients at ICU admission before ARDS and eventually irreversible/persistent respiratory failure develops.

## Additional file

**Additional file 1.** Death from all causes according to level of surfactant protein D. Y-axis is cummulative risk of death. Bold line is for patients with low surfactant protein D; stipulated line is for patients with high surfactant protein D. Grey areas are 95% confidence intervals.
