# Supplementary figures and images for: Prediction of non-recovery from ventilator-demanding acute respiratory failure, ARDS and death using lung damage biomarkers: data from a 1200-patient critical care randomized trial
Source: Ann Intensive Care. 2016 Nov 21;6:114. doi: 10.1186/s13613-016-0212-y (PMC5118375; doi:10.1186/s13613-016-0212-y)

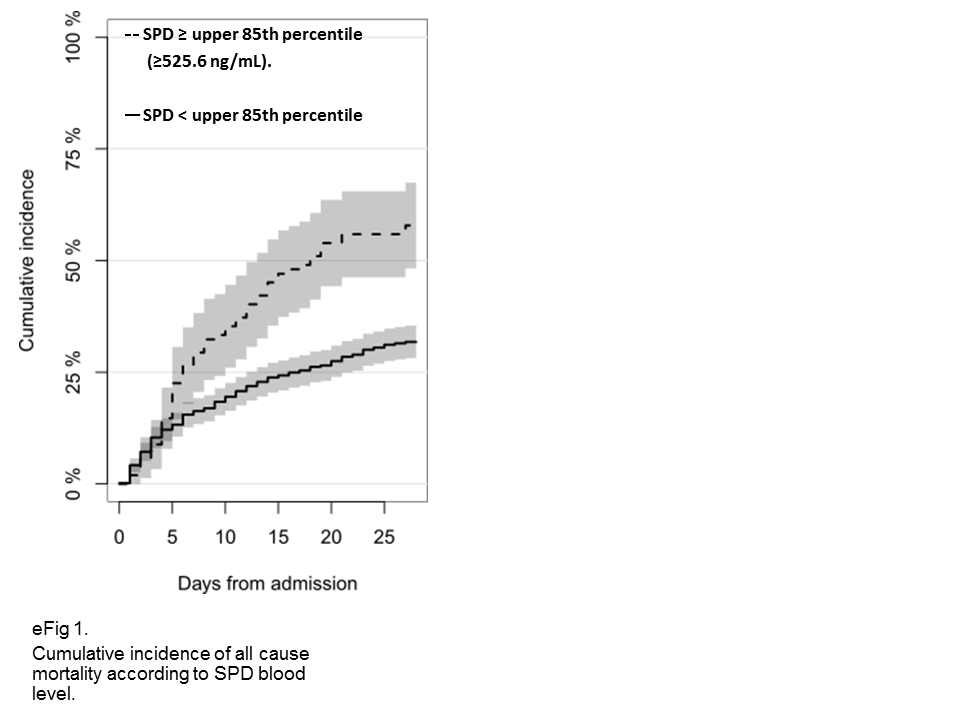

Supplement: Supplementary file 1 — Additional file 1. Death from all causes according to level of surfactant protein D. Y-axis is cummulative risk of death. Bold line is for patients with low surfactant protein D; stipulated line is for patients with high surfactant protein D. Grey areas are 95% confidence intervals. [file 13613_2016_212_MOESM1_ESM.tif]
